# Emotion-focused vs. cognitive interventions of schema therapy for borderline personality disorder: effects on neural emotion regulation networks - study protocol

**DOI:** 10.1186/s40479-025-00311-5

**Published:** 2025-11-13

**Authors:** Stefan Smesny, Kerstin Langbein, Marina Krylova, Meng Li, Igor Izyurov, Alexander Gussew, Daniel Güllmar, Martin Walter, Gerd Wagner, Jürgen R. Reichenbach

**Affiliations:** 1 Department of Psychiatry, Jena University Hospital, Philosophenweg 3, Jena, 07743 Germany; 2 Department of Medical Physics, University Hospital Halle (Saale), Halle (Saale), Germany; 3 Institute of Diagnostic and Interventional Radiology, Medical Physics Group, Jena University Hospital, Philosophenweg 3, Jena, 07743 Germany

**Keywords:** Behavioral therapy, Schema therapy, Borderline personality disorder, Functional brain connectivity, Proton MR spectroscopy, Resting-state functional MR imaging, Glutamate, GABA

## Abstract

**Background:**

While the effects of psychotherapy methods are being intensively researched, little is known about the clinical and neurobiological effects of specific psychotherapeutic interventions. This study examines the effects of experiential emotion-focused and cognitive interventions in schema therapy on emotion regulation in borderline personality disorder.

**Methods:**

In a randomized, single-blinded, parallel group design, clinical effects and effects on resting-state functional connectivity in neural emotion regulation networks and neurotransmitter metabolism (Glx/GABA) in key regions of these networks are compared. The 9-week treatment protocol includes emotion-focused interventions such as chair dialogues, imagery rescripting, or mode role-playing in the test condition; these interventions are omitted in the active control condition (dismantling design). Resting-state functional MR imaging (rsfMRI) and MEGA-sLASER 1 H MR spectroscopy in the pregenual cingulate cortex (pgACC), anteromedial cingulate cortex (aMCC), and dorsolateral prefrontal cortex (DLPFC) are performed before and after the therapy interval and 6 months after the end of therapy and compared with the neurobiological parameters of healthy control subjects. The clinical effects are recorded using a comprehensive test battery and specified using the Reliable Change Index (RCI). Clinical and biological data are examined using mixed model analysis both longitudinally and in terms of their interactions.

**Discussion:**

The aim is to show that different psychotherapeutic interventions have different effects on deficits in emotion regulation associated with specific effects on neural emotion regulation networks. This would contribute to a better understanding of the neurobiological effects and mechanisms underlying psychotherapeutic core interventions and to their more targeted use in BPD and other related disorders in the future.

**Trial registration:**

ClinicalTrials.gov Identifier: NCT06367907, Retrospectively registered, April 2024.

**Supplementary Information:**

The online version contains supplementary material available at 10.1186/s40479-025-00311-5.

## Introduction

The term “emotion dysregulation” is often used to characterize a range of behavioral phenomena that are prominent in borderline personality disorder (BPD) and occur in response to negative emotions, such as fear, anger, anxiety, depression, guilt, and shame, with uncontrollable intensity and duration. Patients report being unable to adequatly regulate these emotions or to communicate them appropriately with others [1]. Psychotherapy (PT) for BPD aims to address the generation, experience, the internal appraisal and the regulation of emotions in response to internal or external stimuli or interactions with others. For this reason, different PT concepts using different techniques [2] have been developed (e.g., dialectic-behavioral therapy, DBT [3, 4]; schema therapy, ST [5–7], Metacognitive Interpersonal Therapy, MIT [8], Mentalization-Based Treatment, MBT [9] or Transference-Focused Psychotherapy, TFT [10]). While the clinical efficacy of the various methods has been proven in most cases, including in direct comparisons (meta-analysis by [11, 12]), the focus is shifting more and more to the underlying factors and mechanisms of action (e.g [8, 13, 14]).,, particularly with regard to emotion regulation disorders in BPD [2].

The aim of the planned study is to use schema therapy (ST) as an example to examine important therapeutic techniques that are also used in other psychotherapy methods. ST itself is based on the idea that aversive experiences and frustrations of basic childhood needs (e.g., safety, love, attention, acceptance, or autonomy), in interaction with biological and cultural factors, lead to the development of maladaptive schemas, defined as organized patterns of information processing, comprising thoughts, emotions, scenic core memories, and attentional preferences. When a maladaptive schema is activated, the painful emotions associated with it are also re-experienced. Since more functional ways of coping are not yet available, the automatic coping strategies (capitulation, avoidance, overcompensation) that have emerged from distress in the past are intuitively used to deal with these intense emotions. Although these attenuate aversive emotions, they interfere with the development of adaptive interpersonal and self-regulatory behavior [5, 15]. To better capture and understand quickly changing emotional and bodily experiences and automated coping strategies in the context of schema activation, the Schema Mode Model was developed [15–17]. According to this, a mode describes the current emotional-cognitive-behavioral state, which can change quickly, whereas a schema is rigid and enduring, reflecting a trait. A detailed description of all modes relevant to this study is given by Fassbinder and colleagues [2, 6]. The schema mode model can therefore be used to describe and explain emotional dysregulation in BPD patients particularly well. Applied to the treatment of emotional dysregulation in BPD, the main goal of ST is to help patients understand their core emotional needs and learn how to meet these needs in an adaptive way or to help them deal with frustration when needs cannot be meet. To achieve these goals, mode-specific experiential, cognitive and behavioral interventions are used. ST emphasizes experiential, emotion-focused techniques such as chair dialogue and imagery rescripting but also includes cognitive techniques. A central therapeutic element of ST in the therapist–patient relationship is “limited reparenting”, whereby the therapist acts as a “good parent” within the boundaries of the therapeutic relationship, offering the patient empathy, warmth, protection, care and space for spontaneity and creativity. It serves as an antidote to traumatic experiences and leads to corrective emotional experiences. In addition, it may be necessary to set boundaries for patients and to confront them empathically with the consequences of their behavior and the need for change. We have used the following neuropsychological models to translate these core elements of ST treatment into a research approach.

**Fig. 1:**
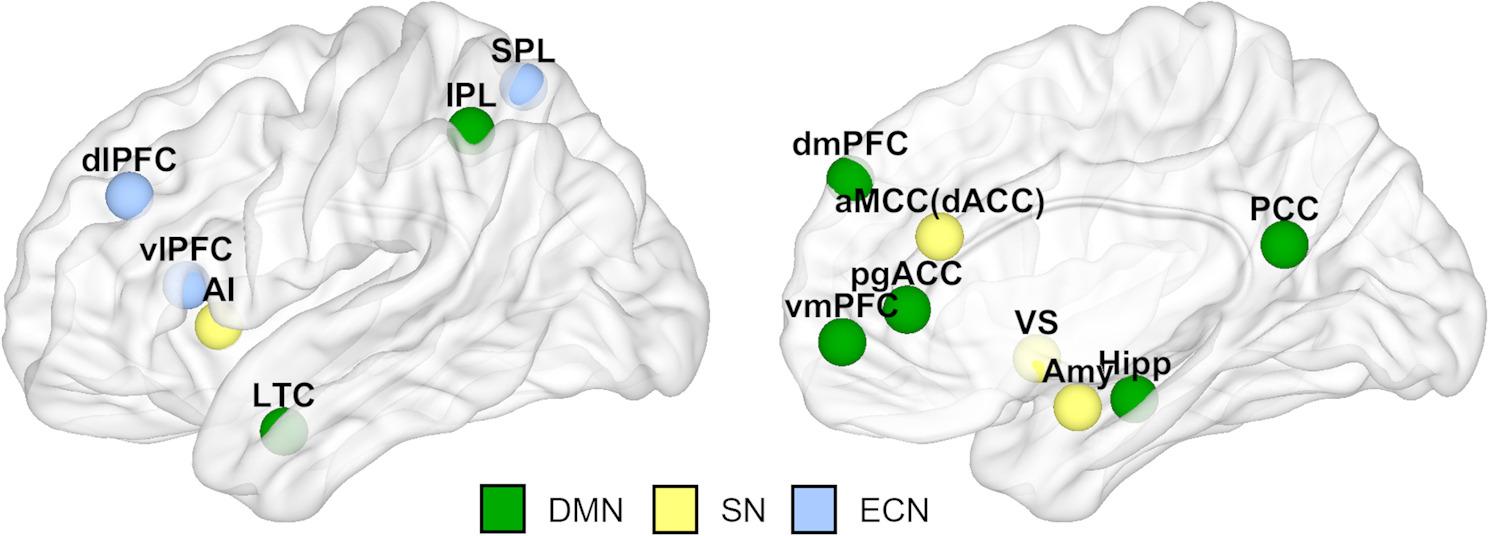
Involved networks and target regions: Illustration of brain regions in the default mode network (DMN) [24, 25], the salience network (SN) and the executive control network (ECN) [26–28]. DMN (marked in green):ventromedial prefrontal cortex (vmPFC), dorsomedial prefrontal cortex (dmPFC), perigenual anterior cingulate cortex (pgACC), posterior cingulate cortex (PCC), hippocampus (Hipp), inferior parietal lobule (IPL), lateral temporal cortex (LTC) SN (marked in yellow): dorsolateral anterior insular cortex (AI), dorsolateral anterior cingulate cortex (aMCC, previous dACC), ventral striatum (VS), Amygdala (AMY) ECN (marked in blue): dorsolateral and ventrolateral prefrontal cortex (dlPFC, vlPFC), superior parietal lobule (SPL)

Gross’ Modal Model of Emotion Regulation is currently the prevailing generic model for describing the emotion-generating process and the response to emotions. The model comprises a situation-*attention*-*appraisal*-*response*-sequence [18–21]. In addition, the Model of Cognitive Control of Emotion (MCCE) [22] identifies brain regions that are suitable for longitudinal observations of these categories. These include (i) the dorsolateral and posterior prefrontal cortex (DLPFC) and inferior parietal regions (IPL), which serve to direct attention to stimulus features relevant to reappraisal and to remember the targets of reappraisal and the content of reappraisal; (ii) dorsal regions of the anterior cingulate cortex (aMCC [previous dACC [23]), which monitor the effects of current reappraisals on emotional responses; and (iii) regions of the ventrolateral prefrontal cortex (VLPFC), which are involved in the selection of goal-directed responses (and inhibition of nontargeted responses) and use information from semantic memory to intentionally select a new, stimulus-appropriate reappraisal in place of the original appraisal of the stimulus. From a network perspective, these regions represent parts of the default mode network (DMN, namely, the IPL) [24, 25], the salience network (SN, namely, the aMCC) [26, 27] and the executive control network (ECN, namely, the DLPFC and VLPFC) [28] (an illustration of the three networks and their respective brain regions is provided in Fig. 1). According to the network perspective, the DMN appears to be most engaged when an individual is left to their own thoughts and during periods of self-reflection, which includes the experiencing of emotions, retrieving autobiographical memories, envisioning the future, and taking perspective [24, 25, 29]. Other investigators relate the involvement of the DMN to automated decisions and actions that govern our daily lives, providing efficient and adaptive responses to environmental demands. This “autopilot” behavior is also memory-based and involves the application of learned rules in predictable behavioral contexts [30, 31]. Thus, the inclusion of the DMN in this study is necessary to understand adaptive brain processing in terms of emotion regulation in the (schema causing) biographical context and change processes induced by psychotherapy.

The amygdala, a key subcortical structure, plays a critical role in emotion generation [20, 32] by perceiving and decoding social-emotional stimuli, particularly those signaling potential threats like fear expressions—and generating biologically relevant information to evaluate the motivational relevance of environmental stimuli in alignment with an individual’s current motivational state and personal goals [33]. A recent meta-analysis by Morawetz et al. [21] provided evidence for the specific involvement of a brain network comprising the amygdala, parahippocampus, and ventromedial prefrontal cortex (VMPFC) in emotional perception and processing. This network seems to be strongly associated with the perception of various emotional stimulus qualities and appears to play a central role in emotional reactivity, the generation of emotional responses, and potentially the appraisal of emotional stimuli.

The network, introduced above as salience network (SN) (namely, the amygdala, aMCC, anterior insula and ventrales striatum) is assumed to be involved in the production of interoception and subjective feelings (e.g., negative affect or pain) as well as coordinating appropriate responses to internal and external events [26, 27, 34]. One of the key features of BPD is an increased sensitivity to recognising negative interpersonal cues that may trigger negative emotions and may be associated with hyperactivity of the SN, thus providing a potential focus for study. Finally, the ECN is activated by tasks that require attention and cognitive control [25]. In the context of psychotherapy, the function of emotion regulation has been translated as “stop and think”, which in neurobiological terms is synonymous with exerting prefrontal cognitive control. This means stopping prepotent behavioral responses, reappraising unwanted emotions, and intentionally modifying behavior with respect to the preferred goal. The intention to stop and think within a therapeutic context requires finding reasons to leave previous (schema-related) patterns and goals while adopting a trusted other’s (the therapist’s) reasons and defining new (own) goals [35].

This study is based on the assumption that there is a dynamic interaction between the SN and the DMN or ECN, whereby the DMN competes with the ECN network. The SN initiates the switching of attentional focus between the lateral frontoparietal ECN and the medial frontoparietal DMN depending on the current task-relevant goals. When outward directed attention and cognitive-emotional processing are required, the ECN is activated, and the DMN is inhibited. Conversley, when inward-directed, self-referential attention and processing are required, the DMN is activated, and the ECN is inhibited [25, 27, 28, 36–38].

ST arose from the clinical experience with cognitive psychotherapy that patients (e.g., with BPD) can question and modify their dysfunctional core beliefs (as part of maladaptive schemata) on the cognitive level but cannot integrate and accept this change emotionally and physically (no de-actualization of the old schemata towards new, more adaptive schemata). Therefore, they stagnate in states of tension and negative emotions because their basic emotional needs (love and secure attachment, autonomy and self-realization, freedom to express themselves and thus be seen and accepted, boundaries, e.g. to learn self-discipline and self-control, or spontaneity and play) remain unsatisfied [2, 15]. In terms of the attractor model, patients are stuck in the old attractor [39–41]. In order to bring about changes at the schema level (away from the old attractor towards a new, more adaptive attractor) and the associated effects on emotion regulation, it seems necessary to address both self-referential, interoceptive (DMN, SN) and knowledge- and insight-based networks (ECN) in the therapy process and to be able to change the focus of attention (SN function) in a therapeutically meaningful and flexible way.

The ST integrates cognitive interventions with a primary focus on the activation of the ECN and experiential interventions with a primary focus on the activation of the SN and DMN, i.e., episodic–emotional biographical content. The present study separates these two therapeutic elements as much as possible in order to investigate the different effects of individual interventions on various networks (ECN, SN, and DMN) and on emotion regulation skills. We combine clinical measurements with two MRI-based methods that complement each other in their focus. 1 H-MR spectroscopy allows the investigation of metabolism and activity levels in structures but does not allow direct conclusions to be drawn about network functions. Resting-state functional MRI (rs-fMRI), on the other hand, uses functional connectivity (RSFC) to provide information on the strength of connections in networks or between structures but does not provide direct information on the type of disruption in the structures themselves. The combination of these two methods allows the co-registration of region-related pathology and the corresponding effects in functional networks and is therefore be expected to provide the greatest gain in information.

The activity of the networks and core structures at the start of the study is used as the starting point for investigating the effects of therapy. Based on previous findings [42, 43], RSFC characteristics of increased activity in limbic structures and corresponding networks (SN, DMN) with reduced activity in the ECN and a specific pattern of Glx and GABA concentrations in the DLPFC, aMCC, and pgACC are expected to be associated with the severity of the emotion regulation deficit.

The study focuses on longitudinal investigations (T0-T1, T0-T2, and T1-T2) of the biological and clinical effects on emotion regulation. The following study questions are investigated: (i) Does the full spectrum of ST interventions (ST-EF) compared to the control condition (ST-AC, omission of emotion-focused interventions, dismantling design) lead to a difference in the clinical efficacy profile on emotion regulation deficits? (ii) Can this difference in clinical efficacy between the two therapy conditions also be represented as a difference in the effect on the network activity of the DMN, SN, and ECN and corresponding core regions?

### Hypotheses

(i) The therapeutic implementation of all ST interventions, including experiential emotion-focused interventions (ST-EF), has different effects on emotion regulation abilities (primary clinical endpoint) than the control condition without these interventions (ST-AC) (see below and Table 1 for outcome parameters and assessment instruments).
(ii)**Table 1:**
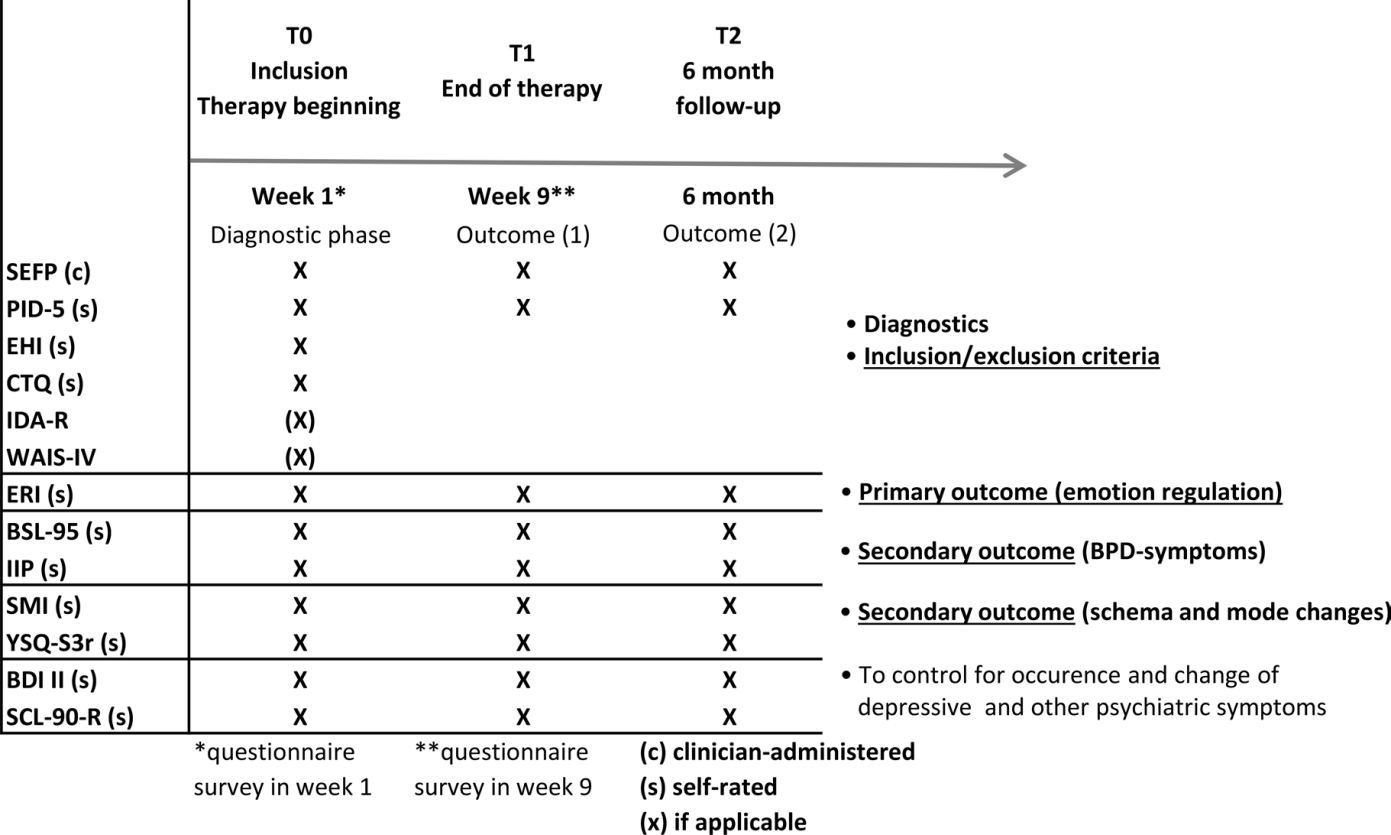
Assessment and assessment intervals
(iii) Under ST-EF treatment (i.e., including emotional activation), a change in activity (RSFC) in the DMN, SN, and ECN is expected, as well as a corresponding change in the Glx/GABA profile in the respective three key regions aMCC (DMN), pgACC (SN), and DLPFC (ECN). In the control condition (ST-AC), however, the most pronounced change is expected in the ECN, with a change in RSFC and the Glx/GABA profile in the DLPFC. Correlations are demonstrated between clinical and neurobiological changes.

## Methods

**Fig. 2:**
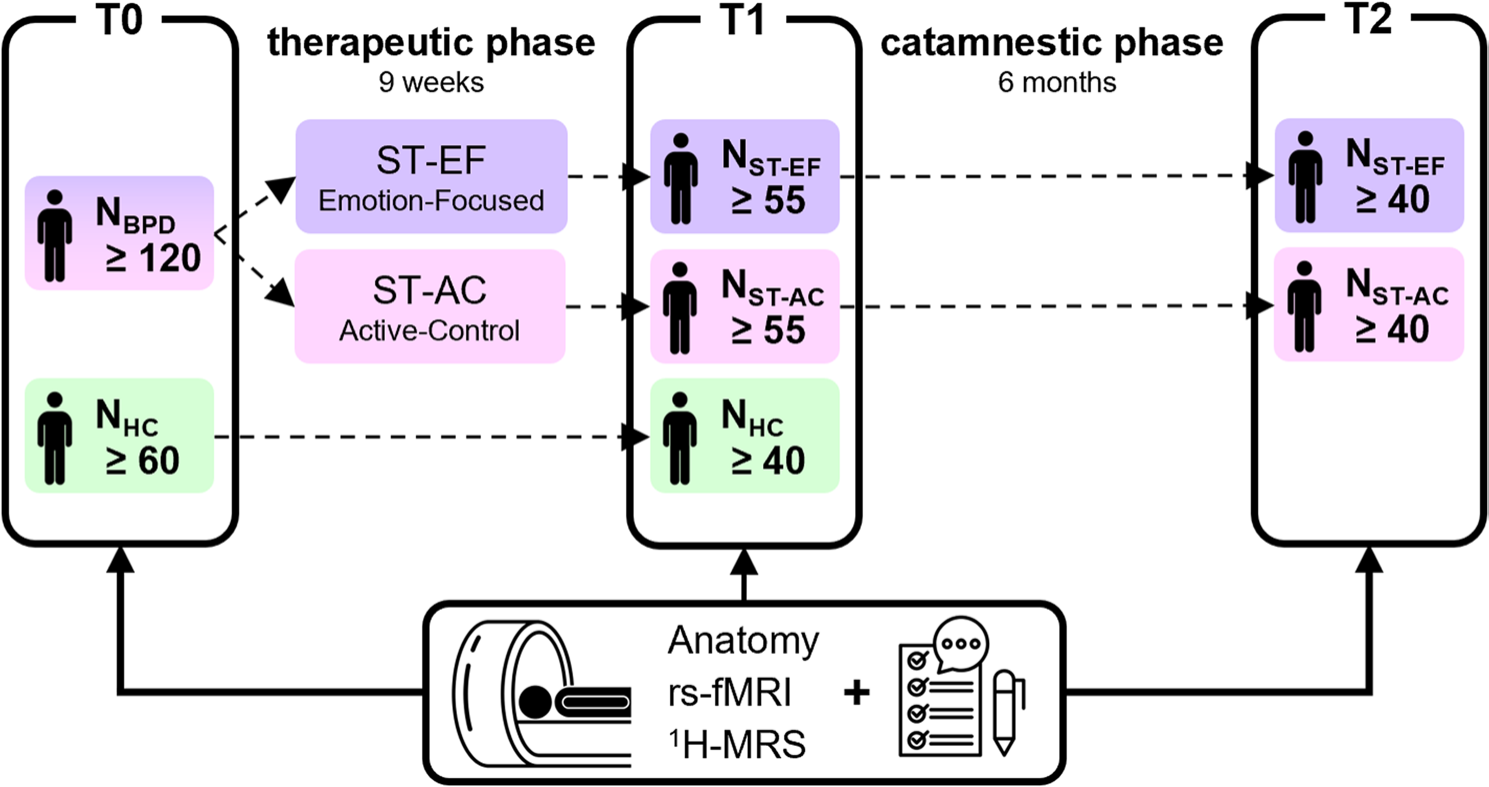
Study design: T0/T1 First therapeutic phase is divided into an emotion-focused (ST-EF) and an active control (ST-AC) condition in terms of emotion-focused interventions of ST. It enables the assessment of differential effects of the experiential therapeutic techniques of ST: *(i)* as compared to the cognitive behavioral techniques serving as control condition (T0/T1), *(ii)* as compared to the influence of time (T0 *vs*. T1 in healthy controls), *(iii)* and as compared to medium-term treatment effects. Marked in grey: Target group sizes as derived from the power calculation, and including possible dropouts in each *group*

We perform a randomized, parallel-group, single-blind dismantling study (the design is illustrated in Fig. 2). Patients are randomly allocated to the ST-EF or ST-AC condition by an independent enrolment manager (senior nurse, not a member of the research team) according to a randomisation list drawn up by an independent statistician. Both conditions (emotion- focused, ST-EF, and active control, ST-AC; see also Table 2) are highly standardized (see below) and active in terms of their expected clinical effect, which is a precondition for positive ethics approval. Researchers will be blinded to participants’ group allocation.

### Sample

Patients are recruited from routine admissions to a psychiatric day clinic specializing in the treatment of personality disorders. In order to meet these therapeutic requirements, schema therapy has been implemented since 2011 and has been successfully adapted to a daily clinical setting [44]. In close cooperation with the Institutes for Schema Therapy in Jena (https://www.schematherapie-jena.de/), Frankfurt (https://schematherapie-frankfurt.de/) and Stuttgart (https://schematherapie-stuttgart.de/), a 9-week therapy programme was developed that includes the core interventions of ST in a condensed and intensified manner and allows the individual allocation of experiential, emotion-focused or cognitive individual and group interventions to the study participants. The criteria for ensuring the quality of the therapy and the conduct of the study are summarized below.

According to a power calculation (more information is given in the supplement), we aim to include at least 120 right-handed patients with an established diagnosis of BPD according to the general and borderline-specific criteria of the alternative DSM-5 Model for Personality Disorders (Section III of DSM 5, p 761ff) [45]. All diagnoses are independently established/confirmed by two board-certified psychiatrists/psychologists (S.Sm., K.L., G.W.) according to the inclusion and exclusion criteria outlined in Table 2. The impairment in personality functioning (criterion A) is measured by the Personality Functioning Scale (LPFS, German Version called Skala zur Erfassung des Funktionsniveaus der Persönlichkeit, SEFP [46–50]. Personality traits characteristic of BPD (criterion B) are assessed by the Persönlichkeits-Inventar für DSM-5 (PID-5 [51–53]).

**Table 2:** Inclusion and exclusion criteria

Inclusion criteria	Exclusion criteria
Patients are eligible if they:	Patients will be excluded if they:
fulfill diagnostic criteria (1.−4. see below) of BPD according to the Alternative DSM-5 Model (Section III of DSM 5, p 761ff)	suffer manifest psychotic disorder according to DSM-5 criteria or have a lifetime history of psychotic disorder (except brief psychotic disorder according to the DSM-5)
are between 18 and 30 years of age	acutely suffer suicidal ideas
are right-handed	have an IQ below 80
are free of any treatment with antipsychotic or mood stabilizing medication (antidepressants or sporadic use of tranquillizers to induce sleep are permitted)	suffer dissociative identity disorder, substance dependence needing clinical detoxification, anorexia nervosa with BMI < 14 or a serious and/or unstable medical illness
have never been in psychotherapy before, in particular schema therapy, dialectic behavioral therapy, cognitive behavioral therapy	are at any risk in terms of MRI investigations
are willing to participate in individual and group therapy	are willing to participate in the study after written informed consent procedure

1. Moderate or more severe impairment in personality functioning as measured by the Personality Functioning Scale (LPFS; official German Version called as Skala zur Erfassung des Funktionsniveaus der Persönlichkeit, SEFP), manifested by characteristic difficulties in two or more of the areas: Identity, Self-direction, Empathy and/or Intimacy (Criterion A)

2. Four or more of the seven pathological personality traits characteristic in BPD (Emotional lability, Anxiousness, Separation insecurity, Depressivity, Impulsivity, Risk taking and Hostility), at least one of which must be Impulsivity (5), Risk taking (6), or Hostility (7) (Criterion B). This criterion is assessed by the Persönlichkeits-Inventar für DSM-5 (PID-5)

3. Evidence of pervasiveness and stability over time (see Criterion C and D)

4. Exclusion of alternative explanations for Personality Pathology (see Criterion E-G)

BPD-specific symptomatology will be assessed using the Borderline Symptom List 95 items (BSL-95) [54, 55], the Emotion Regulation Inventory (ERI)) [56], and the Inventory of Interpersonal Problems (IIP-D) [57, 58]. The intensity of early maladaptive schemas and the respective schema modes are measured by the Schema Mode Inventory 118 Items (SMI) [7, 59, 60] and the Young Schema Questionnaire (YSQ-S3r) [61–63].

General psychopathology will be screened via the Symptom Checklist 1990 Revised (SCL-90-R [64–66]) and Beck’s Depression Inventory-II (BDI-II [67, 68]). The occurrence and type of psychological trauma is assessed via the Childhood Trauma Questionnaire (CTQ [69–71]). Handedness is assessed via the Edinburgh Handedness Inventory (EHI [72]). If applicable, the Wechsler Adult Intelligence Scale - fourth version (WAIS-IV [73]) and the Structured Guide for the Integrated Diagnosis of Adult ADHD (IDA-R [74]) are included in basic diagnostics.

The history of psychotropic medication is carefully recorded. With respect to the use of psychotropic medication, we favor a naturalistic design, so that the use of psychotropic medication does not restrict participation in the study. Depending on the use of psychotropic medication in the overall population, pharmacotherapy will be included in the analysis as a covariate. In addition to the interview questions on substance and alcohol abuse for all participants, patients undergo breath alcohol tests and blood and urine tests for illegal substances (including cannabinoids, amphetamines, cocaine, morphine, and its derivatives). If a test is positive contrary to the patient’s statement at baseline, this is considered an indication of non-compliance and thus an exclusion criterion. Inclusion in the study can only take place if the test is negative. At least 60 healthy volunteers are recruited via newspaper advertisements. They will be screened using a semi-structured interview and self-report measures (SCL-90-R) to assess their current mental status and to exclude individuals with a personal or first-degree family history of psychiatric disorders (including substance abuse) or a history of neurological or major medical conditions that could affect brain function.

The study was approved by the Ethics Committee of the Jena University Hospital. All participants give written informed consent prior to participation in the study.

### Description of therapy conditions with/without experiential emotion-focused interventions

In this study, we wanted to investigate the mode of action of the central therapeutic interventions of ST, which can be divided into the special design of the therapeutic relationship (limited reparenting, empathic confrontation), experiential emotion-focused or cognitive and behavioral techniques [2]. In terms of emotion regulation, each of these intervention techniques has the potential to influence the regulatory chains and networks described above in a specific manner. For example, some interventions evoke intense emotions (child modes) to promote awareness of basic needs, while others aim to establish a “healthy distance” from over-intense emotions to enable appropriate parental care (healthy adult mode). The individualized and flexible use of these intervention techniques aims to help patients overcome their biographically based avoidance automatisms and fears of strong unpleasant emotions and to learn and practice self-care in their current life context. To investigate mechanisms of action, the aforementioned ST intervention techniques are combined into two protocols differing as far as possible in terms of emotion activation (dismantling design) while maintaining their general effectiveness, i.e., the goal of improved emotion regulation should be achievable in both conditions.

### Test conditions

(see Table 3 and 4).

**Table 3:** Therapy protocol for the test condition and active control condition

Treatment protocol		Emotion Focused (ST-EF)	non-Emotion Focused - Active Control (ST-AC)
Week 1-2 (diagnostic phase)			
Dose	Individual sessions	• 2 individual sessions/week, i.e. weekly sessions of 50 minutes with the individual therapist and co-therapist	
	Group sessions	• group education in terms of maladaptive schemata and schema modes (2 x 60 min/week, i.e. 4 modules in 2 weeks)	
	Additionally per week	• Supporting group therapies: ergotherapy (2×60 min), movement therapy (1×60 min)• meetings of group and individual therapists to coordinate the content of treatment	
Focus	Diagnostics and case conceptualization	Patients arrive in their groups • undergo detailed full diagnostic assessment • get acquainted with individual and group therapist • provide biographical information • recognition of key issues • discussion of diagnostic findings including all relevant questionnaires • get knowledge about the maladaptive schemata and the schema mode model • generation of own individual case conceptualization	
Week 3-7 (changing phase)			
Dose	Individual sessions	• 10 sessions (2/week) of 50 minutes with individual therapist • 5 sessions of 50 min (or 10, i.e. 2×25 min/week) with the co-therapist	
	Group sessions	• 2 weekly group sessions of 90 min including max. 10 patients	
	Additionally per week	• Communicative movement therapy (2×60 min) • mindfulness training (2×60 min, incl. daily 30 min to practice) • music therapy (60 min) • time for self-reflection (60 min/day)	• Movement therapy (2×60 min) • Yoga or progressive muscle relaxation, PMR (2×60 min) • drum therapy (1×60 min) • indulcence training (1×60 min)
Focus	Clarification, developing mode awareness, self-empathy and first steps of self-care	• Diagnostic imagination of all relevant biographical core scenes (as often as possible) to clarify the causality of current emotions and bodily experiences and to develop self-empathy	• Consolidation of knowledge about schemas, schema coping, schema modes (child modes, parent modes, coping modes, healthy adult and happy child mode), emotional needs, function of emotions as well as about normal development of children
		• Chair dialogs to clarify function and goals of dysfunctional coping modes in the childhood context, to fight dysfunctional parent modes (punitive or demanding) or to comfort the vulnerable child mode	• Implementation and analysis of self-observation protocols with regard to schema modes, mode chains and success of biographical automatisms in current life
		• Limited reparenting, if vulnerable child or angry child modes are activated signalizing emotional needs	• Generation of mode-specific micro behavioural analysis of situations on the basis of standardized worksheets
		• Imagery rescripting of childhood situations to develop self-empathy and healthy adult self-care using own biographical core situations as a model	• Pro/Contra debates in terms of schema coping modes vs healthy adult function, punitive or demanding parent mode vs healthy adult function etc.
		• Chair dialogs and mode rule plays to clarify inner conflicts in everyday life situations and to develop self-empathy and healthy adult self-care in the current life context	• Cognitive restructuring (searching for good parent sentences instead of old beliefs)
		• Application of new healthy adult strategies in current everyday life situations in the day clinic and at home	• Questioning own intrinsic values instead of parent mode values
Week 8-9 (becoming ones own therapist phase, transfer phase)			
Dose	Individual sessions	• 2 individual sessions/week, i.e. weekly sessions of 50 minutes with the individual therapist and co-therapist	
	Group sessions	• 2 weekly group sessions of 90 min including max. 10 patients	
	Additionally per week	• Communicative movement therapy (2×60 min) • mindfulness training (2×60 min, incl. daily 30 min to practice) • music therapy (60 min) • time for self-reflection (60 min/day)	• Movement therapy (2×60 min) • Yoga or progressive muscle relaxation, PMR (2×60 min) • drum therapy (1×60 min) • indulcence training (1×60 min)
Focus	Consolidation of patients own experiences with new self-care and management strategies	• Development of own values and functional goals on the basis of own emotional needs	• Development of own values and functional goals on the basis of own emotional needs
		• Imaginary work for practicing healthy adult solutions in emotionally activated or potential trigger situations in the patients current and future life	• Preparation for future challenging situations using worksheets
		• Role plays and mode role plays within the session to anticipate challenging or risk situations in the patients current life or future	• Implementation and monitoring success of BEATE-Steps in order to further improve self-care and management
		• Implementation and monitoring success of BEATE-Steps in order to further improve self-care and management

**Table 4:** Dose of psychotherapy input in the test and control condition

Treatment dose of psychotherpy			ST-EF and ST-AC (equally)	sum therapeutic hours	sum therapeutic min
Diagnostic phase	2 Wo	Individual therapy	2 x 50 min	2	100
		Individual co-therapy	2 x 50 min	2	100
		group therapy	4 x 60 min	4	240
Chanching phase	5 Wo	Individual therapy	10 x 50 min	10	500
		Individual co-therapy	5 x 50 min	5	250
		group therapy	10 x 90 min	20	900
Transfer phase	2 Wo	Individual therapy	2 x 50 min	2	100
		Individual co-therapy	2 x 50 min	2	100
		group therapy	4 x 90 min	8	360
		Individual therapy	sum	23	1150
		group therapy	sum	32	1500
		therapy in total		55	2650

#### Experimental arm:

 ST-EF (Schema Therapy – Emotion-Focussed), 9-week treatment protocol. In this test condition, emotion-focused interventions (limited reparenting, imagery rescripting, chair dialogues, mode role play, etc.) are used to work primarily at the experiential level.

#### Active comparator:

 ST-AC (Schema Therapy – Active-Control), 9-week treatment protocol. In the control condition, cognitive interventions (education, guided observation using schema-memo, microanalysis of situations, pro/contra debates, guided training, etc.) are used to work primarily at the level of clarification and understanding.

All patients included in the study will be treated by the same treatment team, i.e., the same individual and group therapists, regardless of which group they belong to. All treatments will take place in a day clinic setting, i.e., daily between 8:30 a.m. and 4:00 p.m. Any medication already prescribed (e.g., an SSRI) will be continued and only changed if clinically necessary.

### Quality criteria

- All individual and group therapists (*n* = 4, all psychological psychotherapists) are trained at the same institute for schema therapy (IST-J) according to the ISST criteria (https://www.schematherapysociety.org/Certification-Menu-2), have been part of the day clinic team for at least 5 years, and are not part of the research team.
- All therapists are supervised weekly by internal (S. Smesny) and external (M. Valente, E. Roediger) certified ST trainers.
- Two team meetings (1 × 1.5 h/week) are held weekly with all individual and co-therapists involved in the treatment, during which the therapeutic progress of each patient is evaluated in detail.
- The day clinic is located in the immediate vicinity of the university hospital, so that in the event of a serious crisis (e.g., suicidal), all care options of a university hospital with maximum care and a mandatory care mandate for the city of Jena are immediately available.
- If deterioration or complications occur in accordance with the exclusion criteria (in particular suicidal crises, increasing self-harm, behavior that jeopardizes therapy, such as alcohol or substance use, deterioration in team-patient contact, etc.) that cannot be constructively overcome during the therapy process, the patient is transferred to guideline-compliant routine care, exactly as required by the medical situation.
- Study patients are randomly assigned to individual and co-therapists.
- The progress of psychotherapy (FEP-2) and negative effects of PT (INEP) are monitored.
- The treatment effects are evaluated by blinded interviewers, i.e., members of the research team who are blinded to the group affiliation of the participants.
- All therapy sessions are video-recorded to enable independent assessments/ratings of the intensity of the patients’ emotional activation and clarification of any crises.

### Clinical outcome measures

#### Primary clinical endpoint: 

The core question of this study is defined as changes in emotion regulation abilities as the primary clinical endpoint, which will be assessed using the corresponding BSL scores of the Borderline Symptom List 95 items (BSL-95) [54, 55] and the Emotion Regulation Inventory (ERI) [56], with the ERI following the process model of emotion regulation proposed by Gross [18].

#### Primary neurobiological endpoint:

 To investigate changes in network activity, resting state functional connectivity (RSFC) in the DMN, SN, and ECN is measured before and after psychotherapy using resting state fMRI. As an expression of a change in the activity of key regions of each network, the Glx/GABA ratio in the aMCC (DMN), pgACC (SN), and DLPFC (ECN) is recorded using 1 H-MRS. An increase in Glx or a decrease in GABA (i.e., an increase in the Glx/GABA ratio) is interpreted as an expression of increased activity.

### MR-based data acquisition and investigation protocol

All scans were acquired via a 3 Tesla Siemens Prisma fit (Siemens, Erlangen, Germany) whole-body MRI scanner (Syngo MR E11 software) and a 64-channel head array receive coil. The protocol begins with a Siemens AutoAlign scout localizer scan (TR = 3.15 ms, TE = 1.37 ms, flip angle = 8°, 128 contiguous sagittal slices, FoV 260 mm, voxel resolution 1.6 × 1.6 × 1.6 mm^3^; acquisition time 0:14 min) to standardize the orientation and positioning of the imaging field of view.

#### Structural measurements: 

First, structural T1-weighted images are acquired via a magnetization-prepared rapid gradient echo (MPRAGE) sequence (TR = 2400 ms, TE = 2.22 ms, flip angle = 8°, 208 contiguous sagittal slices, FoV = 256 mm, voxel resolution = 0.8 × 0.8 × 0.8 mm^3^; acquisition time = 6:38 min).

##### Resting-state fMRT measurements:

 Next, functional T2*-weighted images are acquired via a multiband gradient echo planar imaging (EPI–EPFID) sequence (TR = 1580 ms, TE = 30 ms, flip angle = 90°, 84 interleaved sagittal slices, multiband acceleration factor = 4, FoV 210 mm, voxel resolution 2 × 2 × 2 mm^3^, acquisition time 12:00 min).

#### Spectroscopic measurements: 

T1-weighted structural images are used to place voxels in the pgACC (20 × 30 × 20 mm^3^), aMCC (30 × 20 × 20 mm^3^) and DLPFC (30 × 20 × 20 mm^3^) regions for MRS data collection. The voxels are placed using neuroanatomical landmaks via procedures described by Dou et al. [75] and Pommier et al. [76]. To ensure consistency in MRS voxel placement, all the scans were performed by the same person.

GABA-edited 1 H-MRS scpecta were acquired via the MEGA semi-LASER (MEscher-GArwood Semi-Localization by Adiabatic Selective Refocusing) sequence obtained from the University of Minnesota [77, 78] (TR = 3000 ms, TE = 68.8 ms, 128 pairs of shots, 2048 data points, spectral width = 2 kHz; acquisition time 13:10 min). To limit the amount of frequency drift, the MRS acquisition was broken into 4 blocks of 32 averages, and a system frequency adjustment was performed prior to the start of each acquisition block. Editing pulses are applied at 1.9 ppm (“ON”) and 7.5 ppm (“OFF”) interleaved with the averages. Water suppression was achieved with VAPOR (variable power with optimized relaxation delays) [79] interleaved with outer volume suppression pulses (OVS). Local B0 shimming was performed via the FAST(EST)MAP (fast automatic shimming technique with echo-planar signal trains utilizing mapping along projections) method [80]. In addition to metabolite spectra, two more reference scans are acquired from the same VOI for eddy current correction (with the water-suppressed RF pulses switched off) and for absolute metabolite concentration quantification, using the same parameters as the water-suppressed spectra (8 averages each).

In addition, photoplethysmography (PPG) and respiration recordings are performed during the entire MRI acquisition at a sampling rate of 500 Hz via two SIEMENS modules supplied with an MRI scanner (Physiological Pulse Unit and Physiological ECG/Respiratory Unit, Siemens Medical Solutions Erlangen, Germany).

### Data evaluation and analysis

The SPSS software package (IBM Corp. released 2013, IBM SPSS Statistics for Windows, Version 25.0. Armonk, NY, USA) and R (version 4.4.0) will be used to perform the following statistical tests to verify (or falsify) the previously formulated hypotheses, as well as visualize the results.

### Cross-sectional analysis of baseline (T0) MRI data

#### For rs-fMRI data: 

Both seed-based and network-based correlation analysis will be used to examine the whole-brain RSFC patterns of the regions central to this study, i.e., the amygdala, pgACC, aMCC and DLPFC, and their corresponding canonical networks, the SN, DMN and ECN. Analysis of variance (ANOVA) will be used to test for RSFC differences between patient groups and controls.

#### For ^1^H-MRS data:

Analysis of variance (ANOVA) will be used to test for group differences in the local Glx and GABA concentrations and the Glx/GABA ratio in the pgACC, aMCC and DLPFC between patients and controls.

#### Associations between rs-fMRI and ^1^H-MRS data:

A linear mixed model analysis will be performed to investigate differences in the associations between RSFC in the SN, DMN, and ECN networks and Glx or GABA concentrations in the pgACC, aMCC and DLPFC between patients and controls [81].

### Follow-up (T0-T1, T1-T2, and T0-T2) analysis of treatment effects on clinical endpoints

#### Assessment of changes in emotion regulation:

Assessment of changes in emotion regulation: Under both therapeutic conditions, improvement in emotion regulation according to ERI and BSL-95 is estimated using the RCI, the Reliable Change Index (RCI) [82, 83]. The RCI encompasses changes at the individual level in the context of the changes observed for the entire sample by dividing the difference between the pre- and post-treatment values by the standard error (which includes not only the standard deviation of the measure but also the reliability coefficient) [84, 85]. A change is considered reliable (i.e., not an expression of measurement error) if the RCI is greater than 1.96.

### Follow-up (T0-T1, T1-T, and T0-T2) analysis of treatment effects on MRI target parameters

*For rs-fMRI and 1 H-MRS data*: Using analysis of variance (ANOVA) for repeated measurements, the effects of each therapy condition (ST-EF or ST-AC) or the non-specific effects over time (healthy subjects) on RSFC, Glx, and GABA over time between (T0-T1, T0-T2, T1-T2) (group by time interaction) are examined. In a first step, this analysis is performed separately for both treatment groups and with “group_patients vs. control” defined as the between-subject variable.

In a second step, the difference between the two treatment conditions is examined by inserting the factor “group_ST-EF vs ST-AC” as an between-subject variable into the above model. Covariance analyses are also used to examine possible treatment effects on associations between RSFC and metabolic parameters as well as differences between treatment groups.

#### For clinical and biological outcome parameters:

A linear mixed model analysis will be used that includes MRI data (RSFC, Glx, GABA) that have been significantly affected by therapy (T0-T1, T0-T2, T1-T2) and those emotion regulation scores that have reached the highest RCIs. The detected significant interactions are compared between the ST-EF and ST-AC treatment conditions.

## Discussion

The methodologically combined (rsfMRI + 1 H-MRS) neuroimaging study described here aims to investigate the neurobiological effects of specific psychotherapeutic interventions of schema therapy on known key structures and networks of emotion regulation in patients with BPD. The biological effects of therapy will be related to the clinical effects in terms of emotion regulation and, secondarily, to other core symptoms of BPD. The hypothesis is that experiential, emotion-focused interventions have different effects than cognitive interventions and that both contribute in different ways to the clinical change process of improving emotion regulation.

This study is part of a necessary development in psychotherapy research [42], which is also being followed by other groups [86–89] to think about psychotherapy not only in terms of established, manualized procedures, but in terms of specific interventions that should be combined in a meaningful way based on biological findings and applied with individual goals in mind.

This study is expected to provide insights into the influencing factors and mechanisms of psychotherapy that are not only relevant to schema therapy in particular, but may also be significant for psychotherapy in general, as both interventions examined are also used in other psychotherapy methods. The study thus deliberately shifts the focus from a priori defined psychotherapy methods to the investigation of specific core interventions that are used in various psychotherapy methods and contribute to corresponding neurobiological effects.

Deficits in emotion regulation are a key issue in patients with BPD, which is why this group is a primary target group for investigation in this study. However, similar deficits are also common in other patient groups, such as patients with chronic depressive disorders and anxiety disorders. These groups are often treated with schema therapy in combination with other psychotherapy methods aimed for improving emotion regulation. Therefore, the results of this study may extend beyond BPD patients and have valuable implications for the treatment of a broader spectrum of disorders characterized by deficits in emotion regulation.

The study undoubtedly has its strengths and weaknesses. Its strengths include its reference to a clear neuropsychological model of emotion regulation, the investigation of established networks and core structures of emotion regulation using MRI-based methods, and the use of a comprehensive clinical test battery related to this core symptom of BPS. The MRI-based examination methods (1 H-MRS and rsfMRI) were chosen to complement each other in terms of their informative value. In addition, the clear definition of the patient group, the defined catchment area, the sophisticated psychotherapeutic examination and treatment setting, and the process focus with a 6-month follow-up are considered strengths of the study. The target group size of the patient population is larger than in previous studies [90, 91] and, based on clinical schema therapy studies, a high compliance rate with regard to follow-up is to be expected [92].

This study favors a naturalistic approach, which has both advantages and limitations. Although efforts are made to minimize potential confounding factors, certain heterogeneities in the patient population cannot be completely avoided with regard to symptom severity, demographic variables, traumatic experiences, comorbidities, and (antidepressant) medication. Therefore, some patients will be medicated, while others will not. Efforts will be made to achieve a comparable proportion of medicated/unmedicated patients in each treatment group to enable co-variance and subgroup analyses. On the other hand, the intent-to-treat approach with regard to medication provides interesting insights into the extent to which medication is considered necessary to support psychotherapy, as permitted by current guidelines. Accordingly, pre-treatment conditions (in terms of amygdala activation see meta-analysis by Schulze [93]) and the effects of the psychotherapy interventions studied on the brain may differ between medicated and unmedicated patients.

Due to the expected heterogeneity in the patient population, a proportion of psychologically traumatized patients will also be included in the study, which is estimated in the literature to be more than 50% of BPD patients [8, 94, 95]. While BPD patients with and without a history of trauma are equally prone to limbic system hyperactivity (Schulze et al.), the two subgroups may differ in terms of their neurobiological preconditions and respond differently to the two treatment arms. Although an additional covariate, the possibility of investigating the effect of psychological trauma is seen more as a resource than a disadvantage of the study. Furthermore, defining psychological trauma as an exclusion criterion would violate the naturalistic design and make the results less representative.

With regard to the expected heterogeneity of the patient group, the depression that occurs very frequently in BPD patients is significant, as no hyperactivity of the limbic system has been described for comorbid BPD patients [93]. Here, too, it is expected that the subgroup sizes will allow a more detailed analysis of the effects of these covariate.

Finally, it should be noted that psychotherapy in a defined setting always acts as an overall experience, i.e., it contains factors that have an effect beyond the intended interventions [96]. Not all of these factors can be identified and recorded. It is therefore possible that biological and clinical effects may be recorded whose causality is not accessible in this study.

## Conclusion

In conclusion, this neuroimaging study takes advantage of the innovations of ST to investigate the influence of specific psychotherapeutic core interventions on networks and key regions of emotion regulation in a naturalistic setting. The results may contribute to a better understanding of the neurobiological effects and mechanisms underlying psychotherapeutic core interventions and to their more targeted use in BPD and other related disorders in the future.

## Supplementary information

Supplementary Material 1

## Data Availability

The data that will underpin the findings of this study are not publicly available for reasons of quantity and sensitivity and can be requested from the corresponding author upon reasonable request. The data is stored in a data repository with controlled access at Jena University Hospital.

## References

[1] Laddis A. (2015): The Pathogenesis and Treatment of Emotion Dysregulation in Borderline Personality Disorder. *ScientificWorldJournal* 2015: 1–11. 10.1155/2015/179276 PMC4563094 26380355

[2] Fassbinder E, Schweiger U, Martius D, Brand-de Wilde O, Arntz A. Emotion regulation in schema therapy and dialectical behavior therapy. Front Psychol. 2016;7:1–16. 27683567 10.3389/fpsyg.2016.01373 PMC5021701

[3] Linehan MM, Wilks CR. The course and evolution of dialectical behavior therapy. Am J Psychother. 2015;69:97–110. 26160617 10.1176/appi.psychotherapy.2015.69.2.97

[4] McMain SF, Links PS, Gnam WH, Guimond T, Cardish RJ, Korman L, et al. A randomized trial of dialectical behavior therapy versus general psychiatric management for borderline personality disorder. Am J Psychiatry. 2009;166:1365–74. 19755574 10.1176/appi.ajp.2009.09010039

[5] Kellogg SH, Young JE. Schema therapy for borderline personality disorder. J Clin Psychol. 2006;62:445–58. 16470629 10.1002/jclp.20240

[6] Arntz A, Jacob GA. Schema therapy in practice. An introductory guide to the schema mode approach. Sussex: Wiley; 2012.

[7] Young JE, Arntz A, Atkinson T, Lobbestael J, Weishaar ME, Van Vreeswijk MF, Klokman J. The schema mode inventory (SMI). New York, NY: Schema Therapy Institute; 2007.

[8] Rossi R, Corbo D, Magni LR, Pievani M, Nicolo G, Semerari A, et al. Metacognitive interpersonal therapy in borderline personality disorder: clinical and neuroimaging outcomes from the CLIMAMITHE study-a randomized clinical trial. Personal Disord. 2023;14(4):452–66. 37227866 10.1037/per0000621

[9] Bateman A, Fonagy P. Randomized controlled trial of outpatient mentalization-based treatment versus structured clinical management for borderline personality disorder. Am J Psychiatry. 2009;166:1355–64. 19833787 10.1176/appi.ajp.2009.09040539

[10] Doering S, Horz S, Rentrop M, Fischer-Kern M, Schuster P, Benecke C, et al. Transference-focused psychotherapy v. treatment by community psychotherapists for borderline personality disorder: randomised controlled trial. Br J Psychiatry. 2010;196:389–95. 20435966 10.1192/bjp.bp.109.070177

[11] Rameckers SA, Verhoef REJ, Grasman R, Cox WR, van Emmerik AAP, Engelmoer IM et al. (2021): Effectiveness of psychological treatments for borderline personality disorder and predictors of treatment outcomes: A multivariate multilevel Meta-Analysis of data from all design types. J Clin Med 10. 10.3390/jcm10235622 PMC8658126 34884324

[12] Stoffers-Winterling JM, Storebo OJ, Kongerslev MT, Faltinsen E, Todorovac A, Sedoc Jorgensen M, et al. Psychotherapies for borderline personality disorder: a focused systematic review and meta-analysis. Br J Psychiatry. 2022;221:538–52. 35088687 10.1192/bjp.2021.204

[13] Stoffers-Winterling JM, Storebo OJ, Simonsen E, Sedoc Jorgensen M, Pereira Ribeiro J, Kongerslev MT, et al. Perspectives on dialectical behavior therapy and Mentalization-Based therapy for borderline personality disorder: same, different, complementary?? Psychol Res Behav Manag. 2022;15:3179–89. 36329713 10.2147/PRBM.S342257 PMC9624210

[14] Dadomo H, Panzeri M, Caponcello D, Carmelita A, Grecucci A. Schema therapy for emotional dysregulation in personality disorders: a review. Curr Opin Psychiatry. 2018;31:43–9. 29120915 10.1097/YCO.0000000000000380

[15] Young J. Schema therapy: a practitioner’s guide. New York: Guilford Press; 2003.

[16] Lobbestael J, Van Vreeswijk MF, Arntz A. An empirical test of schema mode conceptualizations in personality disorders. Behav Res Ther. 2008;46:854–60. 18460405 10.1016/j.brat.2008.03.006

[17] Bamelis LL, Renner F, Heidkamp D, Arntz A. Extended schema mode conceptualizations for specific personality disorders: an empirical study. J Pers Disord. 2011;25:41–58. 21309622 10.1521/pedi.2011.25.1.41

[18] Gross JJ. Emotion regulation: conceptual and empirical foundations. In: Handbook of emotion regulation. New York, NY: Guilford Press; 2015. p. 3–22.

[19] Gross JJ. Emotion regulation: taking stock and moving forward. Emotion. 2013;13(3):359–65. 23527510 10.1037/a0032135

[20] Zhang JX, Bo K, Wager TD, Gross JJ. The brain bases of emotion generation and emotion regulation. Trends Cogn Sci; 2025. 10.1016/j.tics.2025.04.013 40447491

[21] Morawetz C, Riedel MC, Salo T, Berboth S, Eickhoff SB, Laird AR, et al. Multiple large-scale neural networks underlying emotion regulation. Neurosci Biobehav Rev. 2020;116:382–95. 32659287 10.1016/j.neubiorev.2020.07.001

[22] Ochsner KN, Silvers JA, Buhle JT. Functional imaging studies of emotion regulation: a synthetic review and evolving model of the cognitive control of emotion. Ann N Y Acad Sci. 2012;1251:E1-24. 23025352 10.1111/j.1749-6632.2012.06751.x PMC4133790

[23] Vogt BA. *Structural organization of cingulate cortex: areas, neurons, and somatodendritic transmitter receptors.*, in *Neurobiology of Cingulate Cortex and Limbic Thalamus.*, B.A. Vogt and M. Gabriel, Editors. 1993, Birkhauser: Boston. pp. 19–70.

[24] Raichle ME, Snyder AZ. (2007): A default mode of brain function: a brief history of an evolving idea. *Neuroimage* 37: 1083-90; discussion 1097-9. 10.1016/j.neuroimage.2007.02.041 17719799

[25] Buckner RL, Andrews-Hanna JR, Schacter DL. The brain’s default network: anatomy, function, and relevance to disease. Ann N Y Acad Sci. 2008;1124:1–38. 18400922 10.1196/annals.1440.011

[26] Craig AD. How do you feel–now? The anterior insula and human awareness. Nat Rev Neurosci. 2009;10:59–70. 19096369 10.1038/nrn2555

[27] Menon V, Uddin LQ. Saliency, switching, attention and control: a network model of insula function. Brain Struct Funct. 2010;214:655–67. 20512370 10.1007/s00429-010-0262-0 PMC2899886

[28] Seeley WW, Menon V, Schatzberg AF, Keller J, Glover GH, Kenna H, et al. Dissociable intrinsic connectivity networks for salience processing and executive control. J Neurosci. 2007;27:2349–56. 17329432 10.1523/JNEUROSCI.5587-06.2007 PMC2680293

[29] Northoff G, Sibille E. Cortical GABA neurons and self-focus in depression: a model linking cellular, biochemical and neural network findings. Mol Psychiatry. 2014;19:959. 25237734 10.1038/mp.2014.108 PMC4436587

[30] Vatansever D, Menon DK, Stamatakis EA. Default mode contributions to automated information processing. Proc Natl Acad Sci U S A. 2017;114:12821–6. 29078345 10.1073/pnas.1710521114 PMC5715758

[31] Smallwood J, Bernhardt BC, Leech R, Bzdok D, Jefferies E, Margulies DS. The default mode network in cognition: a topographical perspective. Nat Rev Neurosci. 2021;22:503–13. 34226715 10.1038/s41583-021-00474-4

[32] Morawetz C, Basten U. Neural underpinnings of individual differences in emotion regulation: A systematic review. Neurosci Biobehav Rev. 2024;162: 105727. 38759742 10.1016/j.neubiorev.2024.105727

[33] Pessoa L, Adolphs R. Emotion processing and the amygdala: from a ‘low road’ to ‘many roads’ of evaluating biological significance. Nat Rev Neurosci. 2010;11:773–82. 20959860 10.1038/nrn2920 PMC3025529

[34] Shackman AJ, Salomons TV, Slagter HA, Fox AS, Winter JJ, Davidson RJ. The integration of negative affect, pain and cognitive control in the cingulate cortex. Nat Rev Neurosci. 2011;12:154–67. 21331082 10.1038/nrn2994 PMC3044650

[35] Patterson CM, Newman JP. Reflectivity and learning from aversive events: toward a psychological mechanism for the syndromes of disinhibition. Psychol Rev. 1993;100:716–36. 8255955 10.1037/0033-295x.100.4.716

[36] Fox MD, Snyder AZ, Vincent JL, Corbetta M, Van Essen DC, Raichle ME. The human brain is intrinsically organized into dynamic, anticorrelated functional networks. Proc Natl Acad Sci U S A. 2005;102:9673–8. 15976020 10.1073/pnas.0504136102 PMC1157105

[37] Uddin LQ, Kelly AM, Biswal BB, Castellanos FX, Milham MP. Functional connectivity of default mode network components: correlation, anticorrelation, and causality. Hum Brain Mapp. 2009;30:625–37. 18219617 10.1002/hbm.20531 PMC3654104

[38] Menon V. The triple network model, insight, and large-scale brain organization in autism. Biol Psychiatry. 2018;84:236–8. 30071947 10.1016/j.biopsych.2018.06.012 PMC6345251

[39] Stroh A, Schweiger S, Ramirez JM, Tuscher O. The selfish network: how the brain preserves behavioral function through shifts in neuronal network state. Trends Neurosci. 2024;47:246–58. 38485625 10.1016/j.tins.2024.02.005 PMC12449835

[40] Heino MTJ, Proverbio D, Marchand G, Resnicow K, Hankonen N. Attractor landscapes: a unifying conceptual model for understanding behaviour change across scales of observation. Health Psychol Rev. 2023;17:655–72. 36420691 10.1080/17437199.2022.2146598 PMC10261543

[41] Recanatesi S, Katkov M, Tsodyks M. Memory states and transitions between them in attractor neural networks. Neural Comput. 2017;29:2684–711. 28777725 10.1162/neco_a_00998

[42] Herpertz SC, Schneider I, Schmahl C, Bertsch K. Neurobiological mechanisms mediating emotion dysregulation as targets of change in borderline personality disorder. Psychopathology. 2018;51:96–104. 29672301 10.1159/000488357

[43] Schmahl C, Niedtfeld I, Herpertz SC. [Borderline personality: alterations to brain structure and function through psychotherapy]. Nervenarzt. 2018. 10.1007/s00115-018-0587-0. 30094483 10.1007/s00115-018-0587-0

[44] Smesny S, Valente M. Variables setting, gleiche Akzeptanz - Wie schematherapeutische behandlungselemente in stationäre und Tagesklinische settings implementiert Werden Können. Verhaltenstherapie Und Verhaltensmedizin. 2016;37:450–65.

[45] Association) AAP. Washingtin. DC; London: UK American Psychiatric Publishing; 2013.

[46] Bender DS, Morey LC, Skodol AE. Toward a model for assessing level of personality functioning in DSM-5, part I: a review of theory and methods. J Pers Assess. 2011;93:332–46. 22804672 10.1080/00223891.2011.583808

[47] Gamache D, Savard C, Leclerc P, Payant M, Berthelot N, Cote A, et al. A proposed classification of ICD-11 severity degrees of personality pathology using the self and interpersonal functioning scale. Front Psychiatry. 2021;12: 628057. 33815167 10.3389/fpsyt.2021.628057 PMC8012561

[48] Hummelen B, Braeken J, Buer Christensen T, Nysaeter TE, Germans Selvik S, Walther K, et al. A psychometric analysis of the structured clinical interview for the DSM-5 alternative model for personality disorders module I (SCID-5-AMPD-I): level of personality functioning scale. Assessment. 2021;28:1320–33. 33155489 10.1177/1073191120967972 PMC8167914

[49] Kampe L, Zimmermann J, Bender D, Caligor E, Borowski AL, Ehrenthal JC, et al. Comparison of the structured DSM-5 clinical interview for the level of personality functioning scale with the structured interview of personality organization. J Pers Assess. 2018;100:642–9. 30907713 10.1080/00223891.2018.1489257

[50] Spitzer C, Muller S, Kerber A, Hutsebaut J, Brahler E, Zimmermann J. [The German version of the level of personality functioning Scale-Brief form 2.0 (LPFS-BF): latent structure, convergent validity and norm values in the general population]. Psychother Psychosom Med Psychol. 2021;71:284–93. 33694153 10.1055/a-1343-2396

[51] Krueger RF, Derringer J, Markon KE, Watson D, Skodol AE. Initial construction of a maladaptive personality trait model and inventory for DSM-5. Psychol Med. 2012;42:1879–90. 22153017 10.1017/S0033291711002674 PMC3413381

[52] Zimmermann J, Altenstein D, Krieger T, Holtforth MG, Pretsch J, Alexopoulos J, et al. The structure and correlates of self-reported DSM-5 maladaptive personality traits: findings from two German-speaking samples. J Pers Disord. 2014;28:518–40. 24511899 10.1521/pedi_2014_28_130

[53] Montes SA, Sanchez RO. The underlying structure of the personality inventory for DSM-5 (PID-5): a general factor of personality psychopathology. Curr Issues Pers Psychol. 2024;12:79–90. 10.5114/cipp/163182 PMC11129047 38807698

[54] Bohus M, Kleindienst N, Limberger MF, Stieglitz RD, Domsalla M, Chapman AL, et al. The short version of the borderline symptom list (BSL-23): development and initial data on psychometric properties. Psychopathology. 2009;42:32–9. 19023232 10.1159/000173701

[55] Bohus M, Limberger MF, Frank U, Chapman AL, Kuhler T, Stieglitz RD. Psychometric properties of the borderline symptom list (BSL). Psychopathology. 2007;40:126–32. 17215599 10.1159/000098493

[56] König D. Die regulation von negativen und positiven emotionen. Entwicklung des Emotionsregulations-Inventars und vergleich von migränikerinnen Mit kontrollpersonen. Wien: Universität; 2011.

[57] Horowitz LM, Rosenberg SE, Baer BA, Ureno G, Villasenor VS. Inventory of interpersonal problems: psychometric properties and clinical applications. J Consult Clin Psychol. 1988;56:885–92. 3204198 10.1037//0022-006x.56.6.885

[58] Bailey C, Abate A, Sharp C, Venta A. Psychometric evaluation of the inventory of interpersonal problems 32. Bull Menninger Clin. 2018;82:93–113. 29791195 10.1521/bumc.2018.82.2.93

[59] Lobbestael J, van Vreeswijk M, Spinhoven P, Schouten E, Arntz A. Reliability and validity of the short schema mode inventory (SMI). Behav Cogn Psychother. 2010;38:437–58. 20487590 10.1017/S1352465810000226

[60] Reiss N, Dominiak P, Harris D, Knörnschild C, Schouten E, Jacob GA. (2011): Reliability and validity of the German version of the schema mode inventory. Eur J Psychol Assess: 1–8.

[61] Phillips K, Brockman R, Bailey PE, Kneebone II. Young schema questionnaire - short form version 3 (YSQ-S3): preliminary validation in older adults. Aging Ment Health. 2019;23:140–7. 29125326 10.1080/13607863.2017.1396579

[62] Yalcin O, Marais I, Lee CW, Correia H. The YSQ-R: predictive validity and comparison to the short and long form young schema questionnaire. Int J Environ Res Public Health. 2023. 10.3390/ijerph20031778. 36767144 10.3390/ijerph20031778 PMC9914719

[63] Kriston L. Reliability and validity of the German version of the young schema questionnaire – short form 3 (YSQ-S3). Eur J Psychol Assess. 2013;29:205–12.

[64] Derogatis LR, Melisaratos N. The brief symptom inventory: an introductory report. Psychol Med. 1983;13:595–605. 6622612

[65] Schauenburg H, Strack M. Measuring psychotherapeutic change with the symptom checklist SCL 90 R. Psychother Psychosom. 1999;68:199–206. 10396011 10.1159/000012333

[66] Franke GH. *SCL-90-R - Symptom Checkliste von L. R. Derogatis – Deutsche Version – Manual*. 2., vollst. überarbeitete Auflage ed. 2000, Göttingen: Beltz Test GmbH.

[67] Beck AT, Steer RA. (1995): Beck-Depression-Inventar: (BDI). *In: Hautzinger M, Herausgeber, Bailer M, Worall H, Keller F*: Testhandbuch. 2., überarbeitete Auflage.

[68] Kühner C, Bürger C, Keller F, Hautzinger M. Reliabilität und validität des revidierten Beck-Depressions-inventars (BDI-II). Befunde Aus Deutschsprachigen Stichproben. Nervenarzt. 2007;78:651–6. 16832698 10.1007/s00115-006-2098-7

[69] Bernstein DP, Fink L. Childhood trauma Questionnaire - A retrospective self-report. Manual. San Antonio: The Psychological Corporation; 1998.

[70] Bernstein DP, Stein JA, Newcomb MD, Walker E, Pogge D, Ahluvalia T, et al. Development and validation of a brief screening version of the childhood trauma questionnaire. Child Abuse Negl. 2003;27:169–90. 12615092 10.1016/s0145-2134(02)00541-0

[71] Klinitzke G, Romppel M, Hauser W, Brahler E, Glaesmer H. [The German version of the childhood trauma questionnaire (CTQ): psychometric characteristics in a representative sample of the general population]. Psychother Psychosom Med Psychol. 2012;62:47–51. 22203470 10.1055/s-0031-1295495

[72] Oldfield RC. The assessment and analysis of handedness: the Edinburgh inventory. Neuropsychologia. 1971;9:97–113. 5146491 10.1016/0028-3932(71)90067-4

[73] Petermann F. *WAIS-IV. Wechsler Adult Intelligence Scale*. Fourth. Edition ed. Deutschsprachige Adaptation der WAIS-IV von D. Wechsler. 2012, Frankfurt: NCS Pearson.

[74] Retz W, Retz-Junginger P, Römer K, Rösler M. Standardised psychopathological rating scales for the diagnosis of ADHD in adults. Fortschr Neurol Psychiatr. 2013;81:381–9. 23856943 10.1055/s-0033-1335740

[75] Dou W, Palomero-Gallagher N, van Tol MJ, Kaufmann J, Zhong K, Bernstein HG, et al. Systematic regional variations of GABA, glutamine, and glutamate concentrations follow receptor fingerprints of human cingulate cortex. J Neurosci. 2013;33:12698–704. 23904606 10.1523/JNEUROSCI.1758-13.2013 PMC6618546

[76] Pommier B, Vassal F, Boutet C, Jeannin S, Peyron R, Faillenot I. Easy methods to make the neuronavigated targeting of DLPFC accurate and routinely accessible for rTMS. Neurophysiol Clin. 2017;47:35–46. 28202333 10.1016/j.neucli.2017.01.007

[77] Deelchand DK, Berrington A, Noeske R, Joers JM, Arani A, Gillen J, et al. Across-vendor standardization of semi-LASER for single-voxel MRS at 3T. NMR Biomed. 2021;34: e4218. 31854045 10.1002/nbm.4218 PMC7299834

[78] Oz G, Tkac I. Short-echo, single-shot, full-intensity proton magnetic resonance spectroscopy for neurochemical profiling at 4 T: validation in the cerebellum and brainstem. Magn Reson Med. 2011;65:901–10. 21413056 10.1002/mrm.22708 PMC3827699

[79] Tkac I, Starcuk Z, Choi IY, Gruetter R. In vivo ^1^H NMR spectroscopy of rat brain at 1 Ms echo time. Magn Reson Med. 1999;41:649–56. 10332839 10.1002/(sici)1522-2594(199904)41:4<649::aid-mrm2>3.0.co;2-g

[80] Gruetter R, Tkac I. Field mapping without reference scan using asymmetric echo-planar techniques. Magn Reson Med. 2000;43:319–23. 10680699 10.1002/(sici)1522-2594(200002)43:2<319::aid-mrm22>3.0.co;2-1

[81] Li M, Danyeli LV, Colic L, Wagner G, Smesny S, Chand T, et al. The differential association between local neurotransmitter levels and whole-brain resting-state functional connectivity in two distinct cingulate cortex subregions. Hum Brain Mapp. 2022;43:2833–44. 35234321 10.1002/hbm.25819 PMC9120566

[82] Wise EA. Methods for analyzing psychotherapy outcomes: a review of clinical significance, reliable change, and recommendations for future directions. J Pers Assess. 2004;82:50–9. 14979834 10.1207/s15327752jpa8201_10

[83] Jacobson NS, Truax P. Clinical significance: a statistical approach to defining meaningful change in psychotherapy research. J Consult Clin Psychol. 1991;59:12–9. 2002127 10.1037//0022-006x.59.1.12

[84] Atkins DC, Bedics JD, McGlinchey JB, Beauchaine TP. Assessing clinical significance: does it matter which method we use? J Consult Clin Psychol. 2005;73:982–9. 16287398 10.1037/0022-006X.73.5.982

[85] Hurst H, Bolton J. Assessing the clinical significance of change scores recorded on subjective outcome measures. J Manipulative Physiol Ther. 2004;27:26–35. 14739871 10.1016/j.jmpt.2003.11.003

[86] Seitz KI, Schouler N, Hundertmark J, Wilhelm M, Franz S, Bauer S, et al. Mechanism-based modular psychotherapy versus cognitive behavioural therapy for adolescents and young adults with childhood trauma experiences: study protocol for a feasibility trial within the German center for mental health. BMJ Open. 2025;15: e090476. 40204309 10.1136/bmjopen-2024-090476 PMC11987137

[87] Schramm E, Elsaesser M, Jenkner C, Hautzinger M, Herpertz SC. Algorithm-based modular psychotherapy vs. cognitive-behavioral therapy for patients with depression, psychiatric comorbidities and early trauma: a proof-of-concept randomized controlled trial. World Psychiatry. 2024;23:257–66. 38727062 10.1002/wps.21204 PMC11083959

[88] Schnell K, Herpertz SC. Emotion regulation and social cognition as functional targets of mechanism-based psychotherapy in major depression with comorbid personality pathology. J Pers Disord. 2018;32:12–35. 29388896 10.1521/pedi.2018.32.supp.12

[89] Elsaesser M, Herpertz S, Piosczyk H, Jenkner C, Hautzinger M, Schramm E. Modular-based psychotherapy (MoBa) versus cognitive-behavioural therapy (CBT) for patients with depression, comorbidities and a history of childhood maltreatment: study protocol for a randomised controlled feasibility trial. BMJ Open. 2022;12: e057672. 35820739 10.1136/bmjopen-2021-057672 PMC9277372

[90] Goodman M, Carpenter D, Tang CY, Goldstein KE, Avedon J, Fernandez N, et al. Dialectical behavior therapy alters emotion regulation and amygdala activity in patients with borderline personality disorder. J Psychiatr Res. 2014;57:108–16. 25038629 10.1016/j.jpsychires.2014.06.020 PMC4263347

[91] Schnell K, Herpertz SC. Effects of dialectic-behavioral-therapy on the neural correlates of affective hyperarousal in borderline personality disorder. J Psychiatr Res. 2007;41:837–47. 17064731 10.1016/j.jpsychires.2006.08.011

[92] Arntz A, Jacob GA, Lee CW, Brand-de Wilde OM, Fassbinder E, Harper RP, et al. Effectiveness of predominantly group schema therapy and combined individual and group schema therapy for borderline personality disorder: A randomized clinical trial. JAMA Psychiatry. 2022;79:287–99. 35234828 10.1001/jamapsychiatry.2022.0010 PMC8892362

[93] Schulze L, Schmahl C, Niedtfeld I. Neural correlates of disturbed emotion processing in borderline personality disorder: a multimodal meta-analysis. Biol Psychiatry. 2016;79:97–106. 25935068 10.1016/j.biopsych.2015.03.027

[94] Frias A, Palma C. Comorbidity between post-traumatic stress disorder and borderline personality disorder: a review. Psychopathology. 2015;48:1–10. 25227722 10.1159/000363145

[95] Porter C, Palmier-Claus J, Branitsky A, Mansell W, Warwick H, Varese F. Childhood adversity and borderline personality disorder: a meta-analysis. Acta Psychiatr Scand. 2020;141:6–20. 31630389 10.1111/acps.13118

[96] Stiles WB, Agnew-Davies R, Hardy GE, Barkham M, Shapiro DA. Relations of the alliance with psychotherapy outcome: findings in the second Sheffield psychotherapy project. J Consult Clin Psychol. 1998;66:791–802. 9803698 10.1037//0022-006x.66.5.791

