# Supplementary material for: Emotion-focused vs. cognitive interventions of schema therapy for borderline personality disorder: effects on neural emotion regulation networks - study protocol
Source: Borderline Personal Disord Emot Dysregul. 2025 Nov 13;12:48. doi: 10.1186/s40479-025-00311-5 (PMC12616957; doi:10.1186/s40479-025-00311-5)
Supplement: Supplementary file 1 — Supplementary Material 1 [file 40479_2025_311_MOESM1_ESM.pdf]

Supplement Power-Calculation

The power calculation for this study is based on the group sizes that are most likely to be required to demonstrate the effects of psychotherapy on biological brain parameters.

We base our estimates on an MR spectroscopy study by O'Neill and colleagues (O'Neill et al., 2017), which found a significant decrease in glutamate in the ACC of patients with obsessive-compulsive disorder after cognitive behavioral therapy (CBT). The active control condition of our study (ST-AC) also includes cognitive techniques and could therefore produce similar effects in BPD patients as the study by O'Neill et al. Since we assume that the active condition of the study (ST-EF) has at least the same effects on clinical and neurobiological target parameters, we used the aforementioned study as the basis for our power calculation. Taking into account the reported mean glutamate reduction of 16.2% under psychotherapy, we can assume an effect size (d) according to Cohen of at least 0.7. Using this effect size, an assumed power of 80%, and a significance level of 5%, at least 30 participants per group are required for the planned study. This calculation fits very well with the group sizes of studies that investigated the biological effects of DBT using fMRI. It also takes into account the finding that ST was significantly effective in a controlled design in more than two-thirds of BPD patients (Giesen-Bloo et al., 2006), comparable to corresponding results in DBT studies (e.g., 42% clinical recovery (Bohus et al., 2004)). Our target for the start of treatment is therefore 60 patients per therapy group. At the time of the 6-month follow-up, the target group size is n = 40 in each treatment condition and thus still exceeds the target of the power calculation, even taking into account study dropouts.

Since the reported effect sizes of psychotherapy interventions on spectroscopic MR measurements were smaller than the effects of psychotherapy or medication on RSFC (Fu et al., 2015; Gimenez et al., 2014; McCabe et al., 2011; Posner et al., 2013; Wang et al., 2015; Wang et al., 2016), we based our power analysis on an effect size of d = 0.7, derived from the above-mentioned MRS study by O'Neil et al. and on group sizes from previous fMRI studies on psychotherapy effects (DBT) in BPD (Bohus et al., 2004).

References

Bohus, M., Haaf, B., Simms, T., Limberger, M.F., Schmahl, C., Unckel, C., Lieb, K., Linehan, M.M., 2004. Effectiveness of inpatient dialectical behavioral therapy for borderline personality disorder: a controlled trial. Behaviour research and therapy 42, 487-499.

Fu, C.H., Costafreda, S.G., Sankar, A., Adams, T.M., Rasenick, M.M., Liu, P., Donati, R., Maglanoc, L.A., Horton, P., Marangell, L.B., 2015. Multimodal functional and structural neuroimaging investigation of major depressive disorder following treatment with duloxetine. BMC Psychiatry 15, 82.

Giesen-Bloo, J., van Dyck, R., Spinhoven, P., van Tilburg, W., Dirksen, C., van Asselt, T., Kremers, I., Nadort, M., Arntz, A., 2006. Outpatient psychotherapy for borderline personality disorder: randomized trial of schema-focused therapy vs transference-focused psychotherapy. Arch Gen Psychiatry 63, 649-658.

Gimenez, M., Ortiz, H., Soriano-Mas, C., Lopez-Sola, M., Farre, M., Deus, J., Martin-Santos, R., Fernandes, S., Fina, P., Bani, M., Zancan, S., Pujol, J., Merlo-Pich, E., 2014. Functional effects of chronic paroxetine versus placebo on the fear, stress and anxiety brain circuit in Social Anxiety Disorder: initial validation of an imaging protocol for drug discovery. Eur Neuropsychopharmacol 24, 105-116.

McCabe, C., Mishor, Z., Filippini, N., Cowen, P.J., Taylor, M.J., Harmer, C.J., 2011. SSRI administration reduces resting state functional connectivity in dorso-medial prefrontal cortex. Mol Psychiatry 16, 592-594.

O'Neill, J., Piacentini, J., Chang, S., Ly, R., Lai, T.M., Armstrong, C.C., Bergman, L., Rozenman, M., Peris, T., Vreeland, A., Mudgway, R., Levitt, J.G., Salamon, N., Posse, S., Hellemann, G.S., Alger, J.R., McCracken, J.T., Nurmi, E.L., 2017. Glutamate in Pediatric Obsessive-Compulsive Disorder and Response to Cognitive-Behavioral Therapy: Randomized Clinical Trial. Neuropsychopharmacology 42, 2414-2422.

Posner, J., Hellerstein, D.J., Gat, I., Mechling, A., Klahr, K., Wang, Z., McGrath, P.J., Stewart, J.W., Peterson, B.S., 2013. Antidepressants normalize the default mode network in patients with dysthymia. JAMA psychiatry 70, 373-382.

Wang, L., Xia, M., Li, K., Zeng, Y., Su, Y., Dai, W., Zhang, Q., Jin, Z., Mitchell, P.B., Yu, X., He, Y., Si, T., 2015. The effects of antidepressant treatment on resting-state functional brain networks in patients with major depressive disorder. Hum Brain Mapp 36, 768-778.

Wang, X., Cao, Q., Wang, J., Wu, Z., Wang, P., Sun, L., Cai, T., Wang, Y., 2016. The effects of cognitive-behavioral therapy on intrinsic functional brain networks in adults with attention-deficit/hyperactivity disorder. Behaviour research and therapy 76, 32-39.
